# Expression of Anti-Lipopolysaccharide Factor Isoform 3 in *Chlamydomonas reinhardtii* Showing High Antimicrobial Activity

**DOI:** 10.3390/md19050239

**Published:** 2021-04-23

**Authors:** Anguo Li, Ruihao Huang, Chaogang Wang, Qunju Hu, Hui Li, Xiao Li

**Affiliations:** 1 Shenzhen Key Laboratory of Marine Bioresource & Eco-Environmental Science, College of Life Sciences and Oceanography, Shenzhen University, Shenzhen 518060, China; lianguo2018@email.szu.edu.cn (A.L.); huangruihao2018@email.szu.edu.cn (R.H.); huqunju@szu.edu.cn (Q.H.); lihui80@szu.edu.cn (H.L.); 2 Guangdong Technology Research Center for Marine Algal Bioengineering, College of Life Sciences and Oceanography, Shenzhen University, Shenzhen 518060, China; 3 Guangdong Provincial Key Laboratory for Plant Epigenetics, College of Life Sciences and Oceanography, Shenzhen University, Shenzhen 518060, China; 4 Department of Journal of Shenzhen University Science and Engineering, Shenzhen University, Shenzhen 518060, China; lixiao@szu.edu.cn

**Keywords:** antimicrobial peptide, anti-lipopolysaccharide factor isoform 3, *Chlamydomonas reinhardtii*, heterologous expression, antimicrobial assay

## Abstract

Antimicrobial peptides are a class of proteins with antibacterial functions. In this study, the anti-lipopolysaccharide factor isoform 3 gene (*ALFPm3*), encoding an antimicrobial peptide from *Penaeus monodon* with a super activity was expressed in *Chlamydomonas reinhardtii*, which would develop a microalga strain that can be used for the antimicrobial peptide production. To construct the expression cluster, namely pH2A-Pm3, the codon optimized *ALFPm3* gene was fused with the *ble* reporter by 2A peptide and inserted into pH124 vector. The glass-bead method was performed to transform pH2A-Pm3 into *C. reinhardtii* CC-849. In addition to 8 μg/mL zeocin resistance selection, the *C. reinhardtii* transformants were further confirmed by genomic PCR and RT-PCR. Western blot analysis showed that the *C. reinhardtii*-derived ALFPm3 (cALFPm3) was successfully expressed in *C. reinhardtii* transformants and accounted for 0.35% of the total soluble protein (TSP). Furthermore, the results of antibacterial assay revealed that the cALFPm3 could significantly inhibit the growth of a variety of bacteria, including both Gram-negative bacteria and Gram-positive bacteria at a concentration of 0.77 μM. Especially, the inhibition could last longer than 24 h, which performed better than ampicillin. Hence, this study successfully developed a transgenic *C. reinhardtii* strain, which can produce the active ALFPm3 driven from *P. monodon*, providing a potential strategy to use *C. reinhardtii* as the cell factory to produce antimicrobial peptides.

## 1. Introduction

Nowadays, people are suffering from the multidrug-resistant (MDR) microorganisms caused by the overuse of antibiotics [1,2,3]. The demand of finding alternatives to replace traditional antibiotics is increasing. Antimicrobial peptides (AMPs) are one of the promising candidates due to their positive effect on killing drug-resistant microorganisms [4,5]. AMPs are peptides consisting of 5–300 amino acids and are widely distributed in various organisms [6,7,8,9]. In general, AMPs own a broad-spectrum antimicrobial activity [10,11], anti-cancer cell activity [12,13], and antiviral activity [14]. As one of the known AMPs, anti-lipopolysaccharide factors (ALFs) were firstly identified from the hemocytes of *Limulus polyphemus* [15]. ALFs have the lipopolysaccharide (LPS) binding domain (LBD), which can bind to LPS to exhibit antimicrobial activity. To date, several ALFs have been identified from *Penaeus monodon* [16,17,18]. Among them, ALFPm3 has a wider anti-bacteria spectrum than other ALFs [19,20,21,22]. AMPs are commonly produced in *Escherichia coli* [23,24,25,26,27] or *Pichia pastoris* [28,29] due to its low yield in natural sources. However, the ALFPm3 could hardly be expressed in *E. coli* possibly because of its strong antimicrobial activity [20]. It was reported that the *ALFPm3* gene could be expressed in *P. pastoris* and its protein extracts showed a broad-spectrum antimicrobial activity, especially on Gram-negative bacteria [30]. However, there were still several defects, such as the low yield, higher cost, CO_2_ releasement, and the challenge of rALFPm3 extraction and purification. Additionally, *P. pastoris* expression system will cause excessive glycosylation, which would restrain the activity of ALFPm3 [31,32,33,34], whereas as a model organism of unicellular green microalgae, *Chlamydomonas reinhardtii* can overcome these problems. Currently, *C. reinhardtii* has established the stable genetic transformation systems in nucleus, chloroplast, and mitochondrion [35,36,37,38,39]. The nuclear genome of *C. reinhardtii* has been sequenced [40] and many mutants have been created for molecular biology research or heterologous expression of useful proteins. To date, a lot of functional enzymes [41], antigens [42], antibodies [43], and vaccines [44] have been successfully expressed in *C. reinhardtii*. As a platform for protein production, *C. reinhardtii* has certain advantages, including rapid growth, capability of protein post-translational processing, and expressing a large amount of foreign protein in chloroplasts. Furthermore, it is capable of photoautotrophy using low-cost medium and it would not release environment- or human-unfriendly metabolites and endotoxins [45,46,47].

Considering the benefits of using chlamy to produce AMPs, it would be attractive if we can produce ALFPm3 in *C. reinhardtii*. In this study, the nucleotides of *ALFPm3* gene were optimized according to the codon bias of *C. reinhardtii* and fused with the *ble* gene mediated by FMDV self-cleavage peptide 2A sequence, and then introduced into *C. reinhardtii* by a glass-bead method. Transformants were screened through zeocin resistance, confirmed by PCR detection, and evaluated by antimicrobial activity analysis. Our results showed that ALFPm3 could be efficiently expressed in transgenic chlamy. It is more important that extracts from transgenic chlamy exhibited a strong antimicrobial activity to bacteria, including some pathogenic bacteria and its performance even better than ampicillin. Our results demonstrated that *C. reinhardtii* is suitable for AMPs production, showing its potentiality to be used as a replacement of antibiotics on aquaculture.

## 2. Results

### 2.1. The Design of ALFPm3 Expression Cassette

**Figure 1:**
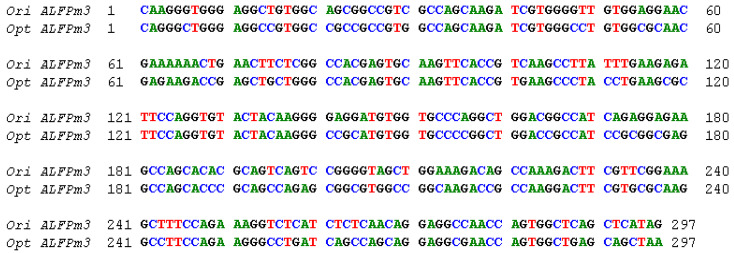
The alignment between the original and codon-optimized *ALFPm3* gene. Ori ALFPm3 refers to the original *ALFPm3* gene, while opt ALFPm3 refers to the optimized *ALFPm3* gene.

**Figure 2:**
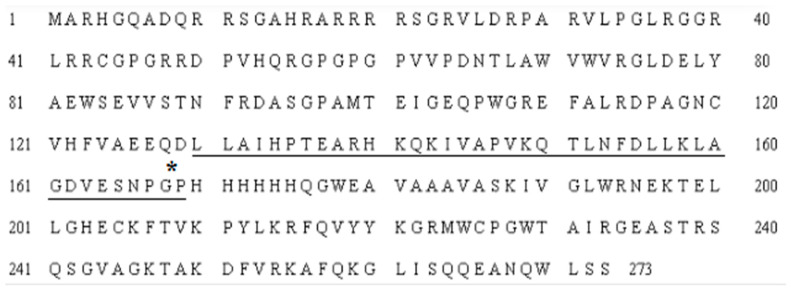
The amino acid sequence of the Ble-2A-Pm3-fused protein. The FMDV 2A sequence has been underlined and the cleavage site is preceded by glycine and proline, which is shown with an asterisk.

**Figure 3:**
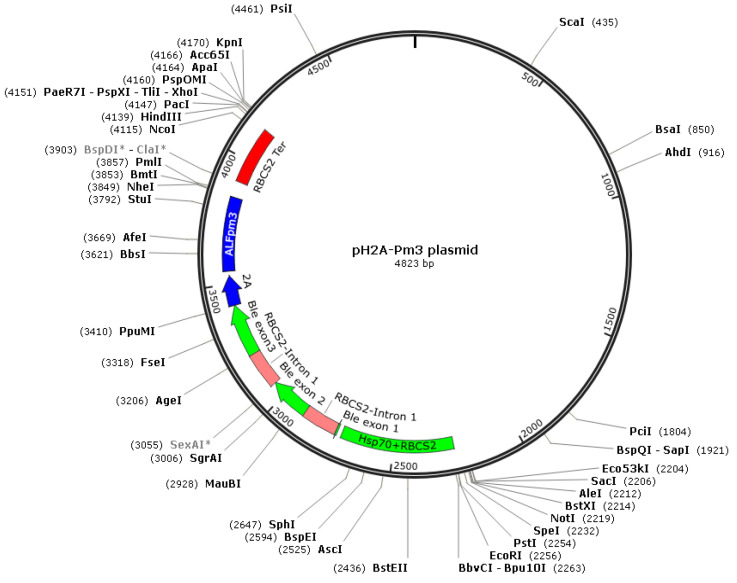
The sketch map of the pH2A-Pm3 plasmid. The restriction sites and their positions are indicated in this figure.

The coding sequence of *ALFPm3* gene from P. monodon (Genbank accession: JQ256520) was optimized according to the codon bias of *C. reinhardtii*. The signal peptide of ALFPm3 composed of 25 amino-acid residues in its N-terminal was removed. After optimization, the GC content of *ALFPm3* gene was increased from 55.89% to 68.37% (Figure 1). The ble gene was fused to the N-terminal of optimized *ALFPm3* gene by Foot and Mouth Disease Virus (FMDV) self-cleavage 2A peptide. FMDV 2A peptide can cleavage between Glycine and Proline to form two independent peptides (Figure 2). Moreover, a 6× His tag was added to the ALFPm3 for protein detection. The designed *ALFPm3* gene expression cassette was then inserted into the pH124 vector resulting in the pH2A-Pm3 plasmid (Figure 3).

### 2.2. The Screening of Transgenic C. reinhardtii

**Figure 4:**
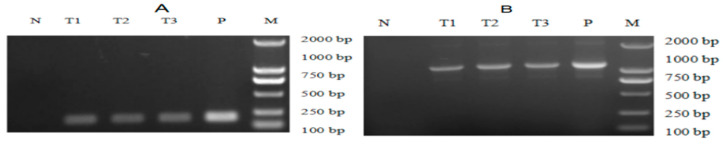
The genomic PCR analysis of transgenic Chlamydomonas. (**A**) PCR analysis for the *ALFPm3* gene. (**B**) PCR analysis for Ble-2A-Pm3-fused gene. N: negative control, P: positive control, T1 to T3: transgenic Chlamydomonas. M: DL 2000 DNA ladder marker.

**Figure 5:**
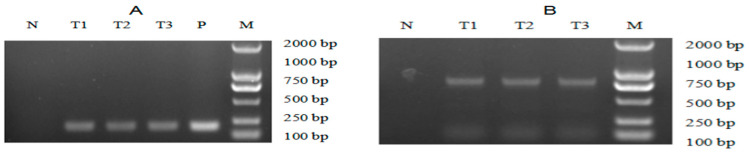
The RT-PCR analysis of transgenic Chlamydomonas. (**A**) RT-PCR analysis for the *ALFPm3* gene. (**B**) RT-PCR analysis for the Ble-2A-Pm3-fused gene. N: negative control, P: positive control, T1 to T3: transgenic Chlamydomonas. M: DL 2000 DNA ladder marker.

The pH2A-Pm3 plasmid was introduced into *C. reinhardtii* using the glass-bead method. After 2 weeks of recovery, the green colonies were visible and transferred to new TAP agar medium containing 8 μg/mL zeocin and 100 μg/mL ampicillin. Here, more than 200 colonies were obtained and the transformation frequency was 3 × 10^−6^. Later, genomic PCR and RT-PCR targeting at both ble and ALFPm3 were conducted to screen positive transformants. PCR results suggested that the non-transgenic *C. reinhardtii* generated no target bands. The specific bands representing the ble and *ALFPm3* genes, respectively, were found in positive transformants, contributing to the positive ratio of 27.27% at the genomic level (Figure 4A). Moreover, the Ble-2A-Pm3-fused expression cassette could be detected in genomic DNA, too (Figure 4B). Furthermore, both optimized *ALFPm3* gene and Ble-2A-Pm3-fused gene in positive transformants were detected in the transcription level (Figure 5). Finally, more than 50 positive transformants were evidenced at both a genomic and transcriptional level and named T-2APm3.

### 2.3. The Analysis of ALFPm3 Protein Production in Transgenic C. reinhardtii

**Figure 6:**
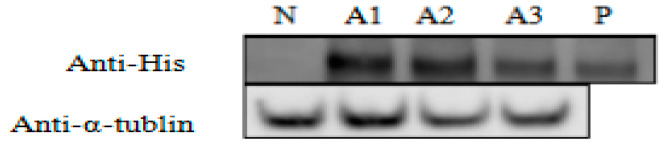
The immunoblot analysis of the ALFPm3 derived from *C. reinhardtii*. Anti-His Tag: protein extracts incubated with mouse anti-His tag antibody for detecting His tag protein. Anti-α-tublin: protein extracts incubated with mouse anti-α-tublin antibody for detecting α-tublin. N: negative control, P: positive control, a protein with 6 × His tag expressed by *E. coli* with a concentration of 3.4 μg/mL. A1 to A3: transgenic Chlamydomonas.

The ALFPm3 peptide with 6 × His tag could be detected by immunoblot using anti-His antibody from total soluble protein (TSP) extracted from T-2APm3 transformants (Figure 6). The molecular weight of ALFPm3 protein was about 12 kD, and the fused protein of BLE-2A-ALFPm3 was 30.64 kD. Our results indicated that the ALFPm3 could separate from BLE-2A-ALFPm3-fused protein by the self-cleavage of FMDV 2A peptide and accounted for approximate 0.35% of the TSP, demonstrating that the *ALFPm3* gene was successfully expressed in *C. reinhardtii*.

### 2.4. The Expression Analysis of ALFPm3 Gene in C. reinhardtii

**Figure 7:**
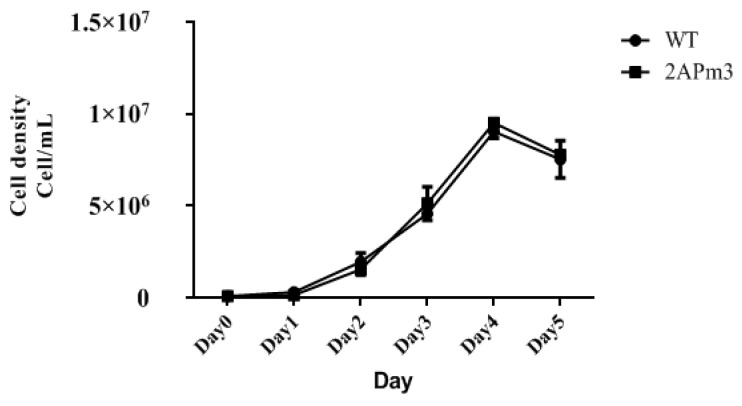
The growth curve of wild type and transgenic chlamydomonas T-2APm3. WT, *C. reinhardtii* CC-849. 2APm3, transgenic chlamydomonas T-2APm3.

**Figure 8:**
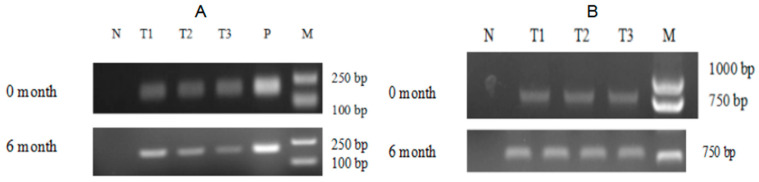
The analysis of ALFPm3 in transgenic Chlamydomonas after 6-month cultivation. (**A**) genomic PCR analysis of the *ALFPm3* gene. (**B**) RT-PCR analysis of the *ALFPm3* gene. N: negative control, P: positive control, T1 to T3: transgenic Chlamydomonas. M: DL 2000 DNA ladder marker.

**Figure 9:**
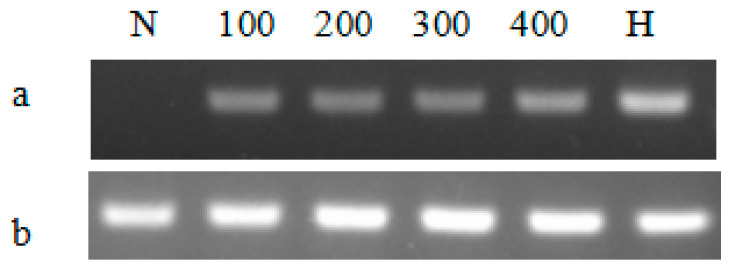
The semi-quantitative RT-PCR analysis of the *ALFPm3* gene in different cultivation conditions. (**a**), RT-PCR analysis for the *ALFPm3* gene, (**b**), RT-PCR analysis for β-actin gene. N: negative control, H: heat shock treatment. 100, 200, 300, and 400 represent 100, 200, 300, and 400 μE·m^−2^·s^−1^ light conditions, respectively.

By comparing the growth curves of T-2APm3 and wild-type, we found that the growth curve of T-2APm3 was identical to that of wild-type (Figure 7), showing that the introduction of the *ALFPm3* gene did not affect the normal growth of *C. reinhardtii*. After 6 months of cultivation, *ALFPm3* gene did not lose or silence, indicating that the target gene could be stably expressed in *C. reinhardtii* (Figure 8). Moreover, heat shock significantly increased the transcription of the *ALFPm3* gene, while the high light increased slightly (Figure 9).

### 2.5. cALFPm3 Showed High Anti-Bacterial Activity

**Figure 10:**
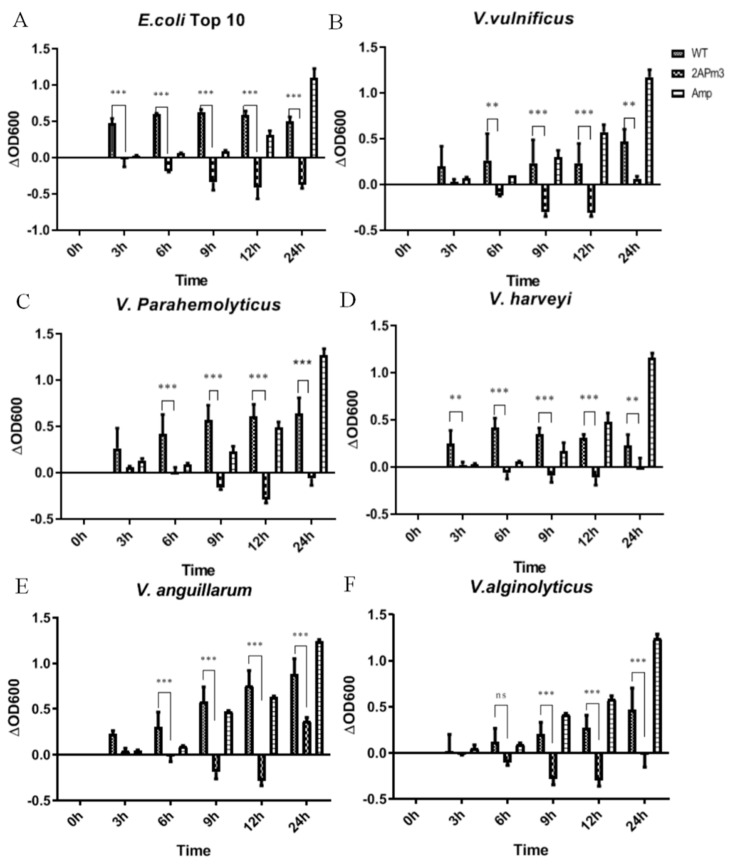
Growth of Gram-negative bacteria under different treatments. Data are statistically analyzed. *** *p* < 0.001. ** *p* < 0.01. ns, no significant. (**A**) *E. coil* Top 10. (**B**) *V. vulnificus*. (**C**) *V. parahemolyticus*. (**D**) *V. harveyi*. (**E**) *V. anguillarum*. (**F**) *V. alginolyticus*.

**Figure 11:**
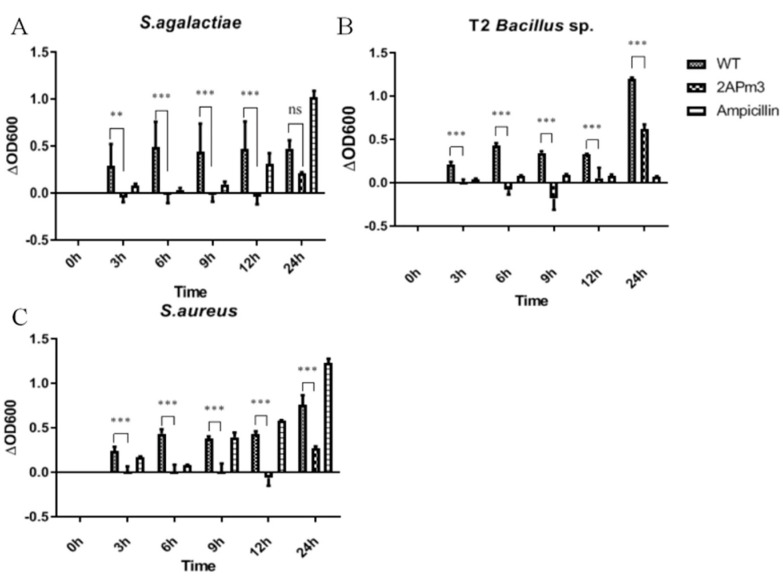
Growth of Gram-positive bacteria under different treatments. Data are statistically analyzed. *** *p* < 0.001. ** *p* < 0.01. ns, no significant. (**A**) *S. agalactiae*. (**B**) *Bacillus* sp. T2. (**C**) *S. aureus*.

After small-scale cultivation, TSP containing ALFPm3 peptide was extracted from T-2APm3 transformants to evaluate the anti-bacterial activity. The results showed that the cALFPm3 at the final concentration of 0.77 μM could significantly inhibit the growth of Gram-negative bacteria such as *E. coli* Top10, *Vibrio vulnificus*, *Vibrio parahaemolyticus*, and *Vibrio alginolyticus* within 12 h (Figure 10). Notably, the antibacterial rate of cALFPm3 against Gram-negative bacteria was 1.4–7.2 times higher than 5 mg/mL ampicillin. Those Gram-negative bacteria, except *V. anguillarum*, hardly grew when treated with cALFPm3 within 24 h (Figure 10). Meanwhile, the inhibition on the Gram-positive bacteria, such as *Streptococcus agalactiae*, *Staphylococcus aureus*, and *Bacillus sp* T2, was also observed within 12 h (Figure 11). For example, the antibacterial rate of cALFPm3 against *S. aureus* was 2.3 times higher than 5 mg/mL ampicillin (Figure 11). The antibacterial activities of cALFPm3 are superior against both Gram-negative bacteria and Gram-positive bacteria and lasted for over 12 h at a concentration of 0.77 μM, which was far beyond the ampicillin (Figure 10 and Figure 11). Hence, our studies provided a promising cALFPm3, which showed a broad antimicrobial spectrum and strong antimicrobial activity.

## 3. Discussion

In this study, we designed an expression cassette of Ble-2A-ALFPm3 to produce the ALFPm3 peptide in *C. reinhardtii*. With the help of 2A peptide, which is a self-cleavage peptide from FMDV [48,49], the ALFPm3 peptide was successfully expressed in *C. reinhardtii* and released from the Ble-2A-ALFPm3-fused protein with a yield of 0.35% of total soluble protein. In order to increase the expression level and prevent gene silencing, the *ble* gene was fused to the N-terminal of the *ALFPm3* gene via the 2A peptide, according to Beth A Rasala et al. [41]. The 2A peptide system has been co-expressed with several proteins at a high level, such as the fluorescent protein targeted to various cellular subcompartments [50], secreted xylanases [41], Cpf1 endonuclease [51], and squalene synthase [52]. Here, the *ALFPm3* gene was co-introduced into *C. reinhardtii* with the *ble* gene. With this expression strategy, we could express the mature peptide of ALFPm3 without its own signal peptide, which was essential for expression in cells. Finally, cALFPm3 could be released from the Ble-2A-ALFPm3 protein and showed a great antimicrobial activity against both Gram-negative bacteria and Gram-positive bacteria. Therefore, this expression strategy was suitable to produce antimicrobial peptides.

Research has indicated that the *E. coli* system could not be used for expressing active AMPs or high-antimicrobial activity peptide due to the lack of post-protein processing [23,24,25,26,27]. The *P. pastoris* system could over-glycosylate AMPs, which reduced the antimicrobial activity [28,29]. Moreover, a lot of materials and energy are required in both *E. coli* and *P. pastoris* system and a large number of harmful gases are produced, including CO_2_ [27,28,29]. On the other hand, with the advantages of rapid growth, high photosynthetic efficiency, and carbon fixation ability, *C. reinhardtii* as a green microalga has been regarded as a novel host for the production of high-value chemicals and recombinant proteins. Although the productivity was not very high in *C. reinhardtii*, cALFPm3 showed high antimicrobial activity at a low concentration. The yield of AMPs in *C. reinhardtii* could reach up to 262 mg/L, as reported by Dong et al. [53]. Moreover, the production of cALFPm3 owned the potentiality to increase its yield with a strong promoter, leaky codon, and Kozak sequence.

It is reported that the ALFPm3 derived from *P. pastoris* exhibited high antimicrobial activity, especially for Gram-negative bacteria at a low concentration (less than 3 μM) and the Gram-positive bacteria at a higher concentration (between 50 mM and 100 mM) [30]. Here, compared with *P. pastoris*-derived ALFPm3, the cALFPm3 showed a higher antimicrobial activity and a long-time effect on the tested bacteria within 24 h at a lower concentration of 0.77 μM. More encouragingly, most of the tested bacteria hardly grew when incubated with 0.77 μM cALFPm3 within 24 h, which is different from the bacteria that were recovered after 9 h when treated with ampicillin. Thus, it is possible to reduce the number of antibacterial substances and obtain better antimicrobial effects by using cALFPm3. Moreover, microalgae are easy to cultivate and processing and is recognized as a safe feed additive, showing its great application in the future.

In this work, an expression cassette of ALFPm3 was designed and expressed in *C. reinhardtii* with high antimicrobial activity. It provides a new way for producing AMPs with high activity and the potential use of AMPs in the aquaculture industry.

## 4. Materials and Methods

### 4.1. Algal Strain and Culture Conditions

*C. reinhardtii* cell wall-deficient strain CC-849 was purchased from the Chlamydomonas Resource Center (USA), and was cultivated mixotrophically in Tris-Acetate-Phosphate (TAP) liquid medium or TAP agar medium contained 100 μg/mL ampicillin (Biosharp, Anhui, China) at 25 °C under a continuous light intensity of 100 μE·m^−2^·s^−1^. Transgenic Chlamy was selected on the TAP agar plate supplemented with 8 μg/mL zeocin (Invitrogen, CA, USA) and 100 μg/mL ampicillin. Algal cells were cultivated until the cell density reached 1 × 10^6^ cell/mL. For the heat shock induction, cells were incubated at 40 °C for 20 min. For high light stress, cells were cultivated under the light intensity of 100, 200, 300, and 400 μE·m^−2^·s^−1^ for 30 min. After each treatment, cells were harvested by centrifugation at 5000× *g* for 5 min.

### 4.2. Plasmid Construction and Genetic Transformation

To improve the transformation frequency and enhance hetero-gene expression, the coding sequence of ALFPm3 (Genbank accession: JQ256520) was optimized according to the codon bias of *C. reinhardtii*. The optimized nucleotides were synthesized by AnHui General Biosystem Company (Anhui, China) and then inserted into pH124 vector to obtain the plasmid pH2A-Pm3 (Figure 3). This vector contains HSP70A-RBCS2 fusion promoter, which can be induced by high light and heat shock. The ble gene was fused to the N-terminal of the *ALFPm3* gene via the FMDV self-cleavage 2A peptide. The constructed plasmid was proliferated in *E. coli* Top10 on an LB agar plate containing 100 μg/mL ampicillin.

The *C. reinhardtii* transformation was processed using glass-bead method according to recommendations [54]. Briefly, the algal cells were cultivated in 100 mL TAP liquid medium in an incubator shaker at the condition of 25 °C, 100 rpm, and continuous light intensity of 100 μE·m^−2^·s^−1^ until the cell density was reached to 1~2 × 10^6^ cell/mL. Cells were harvested by centrifugation at 5000× *g* for 5 min and resolved with 300 μL TAP liquid medium to keep the final density of 2 × 10^8^ cell/mL. Cell cultures were transferred to a new 1.5 mL microcentrifuge tube containing 300 mg glass beads (0.5 mm in diameter) and 2 μg linearized pH2A-Pm3 plasmid. The glass beads were blown gently and vortexed at the highest speed, 2500 rpm, for 25 s. Afterward, the supernatant was transferred to a new microcentrifuge tube with 10 mL TAP liquid medium, cultivated at 25 °C with a light intensity of 90 μE·m^−2^·s^−1^, and shaken continuously at 100 rpm for 22 h. Finally, cells were harvested by centrifugation at 3000× *g* for 5 min, resolved with 100 μL TAP liquid medium, and spread on the TAP agar medium containing 8 μg/mL zeocin and 100 μg/mL ampicillin. They were cultivated at 25 °C in an incubator under a continuous light intensity of 100 μE·m^−2^·s^−1^ until the green colonies were visible. The number of colonies was recorded and counted as the transformation frequency. Transformation frequency = numbers of colonies/total recipient cells.

### 4.3. Genomic PCR and RT-PCR Analysis

The green colonies were copied to a new TAP agar medium containing 8 μg/mL zeocin and 100 μg/mL ampicillin, and cultivated for one week. The positive transformants were selected by genomic PCR as described, targeting the *ble* gene and *ALFPm3* gene [55]. For genomic PCR, 1 × 10^5^ cells were harvested and then resuspended in 50 μL EDTA solution (10 mM, pH 8.0), after which they were incubated in boiling water for 8 min, cooled down on ice for 1 min, and pelleted at 14,000× *g* for 1 min. Finally, one microliter of the extract was used in the 20 μL PCR reaction containing a set of primers (Table 1) and PCR reaction buffer according to the instruction (Mei5 Biotechnology Co., Ltd, Beijing, China). The PCR program was 95 °C for 5 min, 35 cycles of 95 °C for 1 min, 56 °C for 30 s, 72 °C for 30 s, and finally extended at 72 °C for 5 min. For RT-PCR, Total RNA was extracted by RNA fast 200 Kit (Fastagen, Shanghai, China) and 1 μg of the total RNA was used to synthesize the first strand of cDNA by PrimeScript^TM^ RT reagent Kit with gDNA Eraser (Takara, Dalian, China). Two microliter cDNA was used in a 20-μL PCR reaction containing a set of primers (Table 1) and reaction buffer according to the manufacturer’s instructions (Mei5 Biotechnology Co.,Ltd, Beijing, China). For the RT-PCR, β-actin was chosen as an internal control (Table 1). All PCR products were analyzed by 1% agarose gel electrophoresis.

### 4.4. Protein Extraction and Immunoblot Analysis

To evaluate the performance of transgenic *C. reinhardtii* (T-2APm3) on the ALFPm3 production, total protein was extracted and processed on immunoblot analysis. Algal cells were cultivated in TAP liquid medium under constant light intensity of 100 μE·m^−2^·s^−1^ until a cell density of 1~2 × 10^6^ cell/mL was reached. Cells were harvested from one milliliter culture and resuspended with 100 μL lysis buffer (Solution A:Solution B, 3:2, *v/v*) (Solution A: 1M Na_2_CO_3_ 120 μL, 1M DTT 120 μL, deionized water 960 μL, Cock tail 20 μL; Solution B: 5% SDS, 30% Sucrose). After being shaken at 1200 rpm under room temperature for 10 min, cells were frozen-thawed three times in liquid nitrogen with 3 min between each repetition. The supernatant was collected at 14,000× *g* for 10 min at 4 °C for the following western blot analysis. Briefly, 20 μg total protein was separated by 4–20% SDS-PAGE (Genscrip, New Jersey, USA) and then transferred to a PVDF membrane (0.2 μm, Millipore, Massachusetts, USA) activated by 100% methanol. The membrane was then blocked with blocking buffer (5% non-fat dry milk in TBST (10 mM Tris, 166 mM NaCl, pH 7.4, contained 0.05% Tween 20)) for 2 h at room temperature. The membrane was incubated with the mouse anti-His tag antibody (1:8000) (Agrisera, Vännäs, Sweden) in blocking buffer at 4 °C overnight right after washed three times with TBST. Afterward, the membrane was incubated with HRP-conjugated goat anti-mouse IgG antibody (1:10,000) (Beijing Biodragon, Beijing, China) at room temperature for 2 h, and washed with TBST for 3 times. Finally, the membrane was incubated in ECL substrate buffer (Solution A: Solution B, 1:40, *v/v*) (ThermoScientific, MA, USA) at room temperature for 3 min. The chemiluminescence signal was detected by Odyssey·Fc (Gene Company Limited, HK, China). The α-tublin was used as the internal control. The gray values referring protein content were analyzed by Image J software (NIH, Maryland, USA). For calculating the content of target protein, a protein contained 6 × His tag at the concentration of 3.4 μg/mL was used as the calibrator. The content of the target protein was calculated by comparing its gray value with that of calibrator.

### 4.5. Anti-Bacterial Assay

For the anti-bacterial assay, the ALFPm3 peptide was prepared as follows. The transgenic Chlamydomonas T-2APm3 was cultivated in 800 mL TAP liquid medium under a continuous light intensity of 100 μE·m^−2^·s^−1^ until the cell density reached 1~2 × 10^6^ cell/mL. Cultures were centrifuged at 5000× *g* at 4 °C for 5 min, the supernatant was discarded, and pellets were resuspended with 10 mL lysis buffer (Solution A: Solution B is 3:2, *v/v*) (Solution A: 1M Na_2_CO_3_ 120 μL, 1M DTT 120 μL, deionized water 960 μL, Cock tail 20 μL. Solution B: 30% Sucrose) and shaken at 1200 rpm under room temperature for 10 min. Then cells were frozen and thawed three times in liquid nitrogen with 3 min between each repetition. The supernatant contained ALFPm3 peptide was obtained by centrifugation at 14,000× *g* for 10 min at 4 °C, followed by filtration with 0.22 μm filter tip. To investigate the antibacterial activity of peptide extracts, a total of six Gram-negative bacteria (*E. coli* Top 10, *Vibrio vulnificus*, *Vibrio parahemolyticus*, *Vibrio harveyi*, *Vibrio anguillarum*, and *Vibrio alginolyticus*) and three Gram-positive bacteria (*Streptococcus agalactiae*, *Bacillus sp* T2, *Staphylococcus aureus*) were tested. Before the assay, the tested bacteria were cultivated in 5 mL LB liquid medium under 37 °C with shaking at 200 rpm overnight. The 500 μL cultures were added into 5 mL fresh LB liquid medium and cultivated at 37 °C with shaking at 200 rpm for 1 h. Then, the bacteria cultures were diluted by 1000 times using fresh LB medium and 150 μL diluted cultures were mixed with 50 μL ALFPm3 peptide in 96-well plate. The final concentration of ALFPm3 peptide was 0.7669 μM. The extracts from wide type *C. reinhardtii* and 5 mg/mL ampicillin acted as negative and positive control, respectively. Finally, the mixture of bacteria and ALFPm3 was incubated at 37 °C and the OD600 was determined at 0 h, 3 h, 6 h, 9 h, 12 h, and 24 h, respectively. The growth rate (ΔOD600) of the bacteria under different treatments was obtained by comparing the OD600 value with that at 0 h.

### 4.6. Expression Analysis of ALFPm3 in C. reinhardtii Transformants

To observe the expression of ALFPm3 in *C. reinhardtii*, the transgenic Chlamydomonas T-2APm3 was kept constant for 6 months and the total RNA of T-2APm3 was isolated and followed by RT-PCR with primers ALF3-F and ALF3-R. PCR products were analyzed by 1% agarose gel electrophoresis. The growth curves of non-transgenic *C. reinhardtii* and transgenic *C. reinhardtii* were measured to analyze the effect of ALFPm3 on algae growth. They were cultivated in 100 mL TAP liquid medium under continuous light intensity of 100 μE·m^−2^·s^−1^ with shaking. Cell numbers were counted every day via a hemocytometer measurement.

### 4.7. Statistical Analysis

Each experiment was conducted with three independent replicates. GraphPad Prism 5 was used to perform the statistical analysis and generate figures and tables. Student’s t-test was used to calculate the statistical difference between treatments and *p* < 0.05 was considered as showing significant differences.

## Figures and Tables

**Table 1:** Sequence of primers used in this study.

Name	Sequence (5′-3′)	Target Gene
Ble-F	TTAAATCTAGAAAAATGGCCAG	*ble*
Ble-R	GTCCTGCTCCTCGGCCACG	
ALF3-F	AAGTTCACCGTGAAGCCCTAC	*ALFPm3*
ALF3-R	CTGCTCAGCCACTGGTTCGC	
actin-F	ACCCCGTGCTGCTGACTG	*β-actin*
actin-R	ACGTTGAAGGTCTCGAACA	

## Data Availability

Not applicable.
